# P04.03. Primary care providers’ attitudes and beliefs about, and personal use of, complementary and alternative medicine (CAM)

**DOI:** 10.1186/1472-6882-12-S1-P273

**Published:** 2012-06-12

**Authors:** M George, A Johnson, R Pinilla, C Rand

**Affiliations:** 1 University of Pennsylvania School of Nursing, Philadelphia, USA; 2 Johns Hopkins University, Baltimore, USA

## Purpose

To identify primary care providers’ (PCP) attitudes and beliefs about, and personal use of, complementary and alternative medicine (CAM).

## Methods

PCPs who referred patients into a research study about asthma self-management, including CAM, completed three surveys. The modified Integrative Medicine Attitude Questionnaire (m-IMAQ) is a 23-item survey that solicits beliefs about CAM and its role in treatment. The 10-item CAM Health Belief Questionnaire (CHBQ) asks about CAM attitudes and beliefs not included in the m-IMAQ. Lastly, the 30-item Morehouse College Survey of CAM Practices (MCSCAMP) characterizes PCPs personal CAM use. All subjects received a $100 gift card for participation.

## Results

Of the 21 referring PCPs, 14 physicians and two nurse practitioners (NPs) were enrolled. Seven were male (44%); 13 White (81%), two Asian (13%) and one Black (6%). The mean age in years of subjects was 45.7 and mean years in practice was 12.9; all but one was employed full-time. After reverse coding, higher m-IMAQ and CHBQ scores indicate more positive CAM orientation. All 16 providers (100%) endorsed m-IMAQ items: "The spiritual beliefs of patients play an important role in their recovery"; "A strong relationship between patients and their providers is an extremely valuable therapeutic intervention that leads to improved outcomes"; and "In research, measuring quality of life is equally as important as measuring disease-specific outcomes." Only one CHBQ item was endorsed by all: "A patient’s expectations, health beliefs and values should be integrated into the patient care process." The MCSCAMP found prayer/spiritual healing to be the most common CAM for providers’ personal use (50%), followed by massage and acupuncture (19%), music therapy, herbs and meditation (13%).

## Conclusion

PCPs in this sample have a positive CAM orientation uniformly endorsing items related to spirituality, the importance of patient-provider partnerships and quality of life. There was low personal use of CAM other than prayer by providers.

